# Macrophyte assisted phytoremediation and toxicological profiling of metal(loid)s polluted water is influenced by hydraulic retention time

**DOI:** 10.1007/s11356-024-33934-2

**Published:** 2024-06-19

**Authors:** Aqib Hassan Ali Khan, Alberto Soto-Cañas, Carlos Rad, Sandra Curiel-Alegre, Carlos Rumbo, Blanca Velasco-Arroyo, Herwig de Wilde, Alfredo Pérez-de-Mora, Sonia Martel-Martín, Rocío Barros

**Affiliations:** 1 International Research Center in Critical Raw Materials for Advanced Industrial Technologies (ICCRAM), University of Burgos, Centro de I+D+I. Plaza Misael Bañuelos S/N. 09001, Burgos, Spain; 2 Research Group in Composting (UBUCOMP), Faculty of Sciences, University of Burgos, Plaza Misael Bañuelos S/N, 09001 Burgos, Spain; 3 Department of Biotechnology and Food Science, University of Burgos, Plaza Misael Bañuelos, S/N. 09001, Burgos, Spain; 4 Department of Soil and Groundwater, TAUW België Nv, Waaslandlaan 8A3, 9160 Lokeren, Belgium; 5 Department of Soil and Groundwater, TAUW GmbH, Landsbergerstr. 290, 80687 Munich, Germany

**Keywords:** Phytoremediation, Macrophyte, Metal and metalloid contamination, Hydraulic retention time, Toxicity reduction, Phytostabilization, Wetland mesocosm

## Abstract

The present study reports findings related to the treatment of polluted groundwater using macrophyte-assisted phytoremediation. The potential of three macrophyte species (*Phragmites australis*, *Scirpus holoschoenus*, and *Typha angustifolia*) to tolerate exposure to multi-metal(loid) polluted groundwater was first evaluated in mesocosms for 7- and 14-day batch testing. In the 7-day batch test, the polluted water was completely replaced and renewed after 7 days, while for 14 days exposure, the same polluted water, added in the first week, was maintained. The initial biochemical screening results of macrophytes indicated that the selected plants were more tolerant to the provided conditions with 14 days of exposure. Based on these findings, the plants were exposed to HRT regimes of 15 and 30 days. The results showed that *P. australis* and *S. holoschoenus* performed better than *T. angustifolia,* in terms of metal(loid) accumulation and removal, biomass production, and toxicity reduction. In addition, the translocation and compartmentalization of metal(loid)s were dose-dependent. At the 30-day loading rate (higher HRT), below-ground phytostabilization was greater than phytoaccumulation, whereas at the 15-day loading rate (lower HRT), below- and above-ground phytoaccumulation was the dominant metal(loid) removal mechanism. However, higher levels of toxicity were noted in the water at the 15-day loading rate. Overall, this study provides valuable insights for macrophyte-assisted phytoremediation of polluted (ground)water streams that can help to improve the design and implementation of phytoremediation systems.

**Supplementary Information:**

The online version contains supplementary material available at 10.1007/s11356-024-33934-2.

## Introduction

The xenobiotic concentration of metals and metalloids, further referred to as metal(loid)s, poses a severe environmental risk of biomagnification (El-Meihy et al. 2019; Talpur et al. 2020). This is due to the intrinsic capacity of these contaminants, which include their indestructible nature and bioavailability to flora and fauna (Esteves-Aguilar et al. 2023; Manzoor et al. 2016). The contamination of groundwater with metal(loid)s is caused by natural and anthropogenic factors (Iqbal et al. 2021). Natural factors, including aquifer mineral composition, climate, strata geology, water‒rock interactions, weathering, topography, and tidal effects, are known to impact the level of metal(loid)s in groundwater (Khatri and Tyagi 2015). These factors are beyond human control. However, anthropogenic activity also introduces xenobiotic metal(loid)s into the environment (Li et al. 2022). If metal(loid)-contaminated water is discharged into aquatic or territorial systems, it will surely cause deleterious environmental consequences (Ahmad et al. 2023). Hence, reducing metal(loid) xenobiotic levels is vital for preventing any environmental stress. The use of phytoremediation for the removal of legacy and emerging pollutants, including metal(loid)s, is a potential option (Mushtaq et al. 2020; Hussain et al. 2021a).

Phytoremediation has gained popularity due to its ease of operation and maintenance and natural aesthetic appeal (Baloch et al. 2020). When this nature-based solution (NBS) is applied at a commercial scale, there are many other nontangible benefits, including the creation of leisure areas and recreational wellbeing through green spaces (Khan et al. 2024; Raza et al. 2019). Additionally, NBS application can improve microclimates, reduce flood, and storm peaks in urban and preurban surroundings, generate value-added biomass, promote water reuse, and maintain and expand local and migrant biodiversity, which is essentially not possible with commercially available physical and chemical treatment methods (Khan et al. 2019a, 2019b; Curiel-Alegre et al. 2022). One way to assess plant potential for phytoremediation is to study the biochemical and physiological responses of selected plants. For this purpose, plant biomass, root length, plant photosynthetic pigment contents, and enzyme activities (catalase, ascorbate peroxidase, guaiacol peroxidase, and superoxide dismutase) can be assessed (Iqbal et al. 2020). Abrupt fluctuations in plant enzyme activities are well reported as good indicators of plant stress (Saleem et al. 2023). As these enzymes play a significant role in the detoxification of metal(loid)s within the plant body, any such abrupt change can hinder the plant’s capacity to mitigate the negative impact of cellular metal(loid)s (Arshad et al. 2017; Khan et al. 2023a, 2023b). Hence, a potent plant candidate for phytoremediation should show tolerance and minimal changes in physiology and biochemical status (Aftab et al. 2021). Furthermore, the matrix from which the pollution is to be removed also plays a significant role in plant selection. If there is contamination in the soil, it is highly appropriate to use terrestrial plants and trees; however, if it is in the water, then aquatic plants should be used. Like natural wetlands, aquatic plants can be incredibly effective at filtering and cleaning polluted water (Velasco-Arroyo et al. 2024). However, unlike natural wetlands, which can passively receive polluted water, aquatic plants used for water treatment in constructed wetlands require a controlled environment and irrigation to maintain the flow of water and pollutant removal/retention (Martínez-Megías et al. 2024). As the present study focused on the contamination of metals in groundwater, ideal plants would be aquatic, and these amphibious plants are able to thrive in both water and soil environments for an extended period (Enochs et al. 2023). Hence, choosing the most sustainable macrophytes is crucial for large-scale phytoremediation projects to cleanse the contaminated environmental matrices and protection of the flora and fauna at the site.

In the present study, *Phragmites australis*, *Scirpus holoschoenus*, and *Typha angustifolia* were used as model plants to study the impact of metal(loid)s on the biochemical and physiological status of these plants. Even after identifying potential plants and performing phytoremediation of metal(loid)s or any other pollutants from the contaminated matrix, it is possible that in addition to the removal of pollutants/contaminants, the toxicity of treated water is not significantly reduced (Che et al. 2024). As water is a complex matrix, having many physical and chemical parameters that can contribute towards the overall toxicity, removing only metal(loid)s from water is not a key performance indicator (Staszak and Regel-Rosocka 2024). Similar findings were noted by Perotti et al. (2020), who reported that even though hexavalent Cr [Cr(VI)] was significantly reduced (up to 98%), when the concentration of Cr(VI) was 10 mg L^−1^ using in the hairy roots of *Brassica napus*, moderate germination toxicity was noted against *Lactuca sativa* L. even after phytoremediation. Another way to assess toxicity is to use the impact of remediated water on the bioluminescence of the bacterium *Vibrio fischeri*. This bioluminescence assay is much more straightforward and a good way to assess aquatic toxicity (Sigurnjak Bureš et al. 2021). Hence, it can also be proposed that the efficacy of phytoremediation should be assessed by comparing both the reduction of pollutant load/concentration as well as the level of toxicity of the resulting treated effluent*.*

In this regard, it is highly important that to achieve profound acceptance among the scientific community and consultants for the choice of application, phytoremediation must offer both direct and indirect benefits. Most of the previously referred studies focused only on the removal of pollutants from contaminated environmental matrices; however, the toxicity of treated water has not been investigated. The present work aimed to comprehensively assess the potential of three plant species, *Phragmites australis*, *Scirpus holoschoenus*, and *Typha angustifolia*, not only for removing metal(loid)s from groundwater, but also for reducing their overall metal(loid)s toxicity. For these objectives, the ability of the macrophytes to tolerate acute exposure to metal(loid)-polluted water for 7 and 14 days was first assessed, and then, based on these initial findings, the plants were evaluated for their metal(loid) remediation potential and toxicity reduction in a 4-L (L) mesocosm experiment, at varying exposure durations (15 and 30 days) to evaluate the impact of hydraulic retention time (HRT) on their treatment capacity. Furthermore, using the *V. fischeri* bioluminescence assay, the toxicity of treated groundwater was also investigated.

## Materials and methods

### Collection and characterization of polluted groundwater

Polluted groundwater was collected from an old industrial site located in Flanders, Belgium. Groundwater contamination with metals and metalloids (Pb, Zn, Cu, As, and Ni) was caused by former industrial electrolysis activities. Installation was severely damaged at different times in the past century. Consequently, exhausted electrolytes and acids were poured and leaked into the soil and leached into the groundwater. The intended purpose of the treatment using phytoremediation for groundwater was to minimize the environmental impact and ensure compliance with local groundwater standards by comparing the treated water quality to relevant discharge regulations. The metal(loid) concentrations (in µg L^−1^) of the polluted groundwater (PW) were as follows: Ni 127,000, Cu 163,000, Fe 382,000, Zn 72,000, As 300, Cd 2000, and Pb 300 at pH 3.7 and EC 5.3 dS m^−1^, all of which are above the permissible Flemish groundwater sanitation standard threshold values for groundwater (in µg L^−1^): Ni 40, Cu 100, Fe 200, Zn 500, As 20, Cd 5, and Pb 20 [24]. Consequently, compared with those of the sanitation standard, the values of metal(loid)s decreased in the following order: Ni (3182), Fe (1910), Cu (1,632), Cd (441), Zn (143), As (16), and Pb (16).

### Mesocosm preparation for the biochemical profiling of the plants (batch testing)

Plant seedlings of *P. australis*, *S. holoschoenus*, and *T. angustifolia* were purchased from Viveros La Dehesa, Valdeobispo (Spain). The plants were maintained in a plant growth room located at the Universidad de Burgos (Spain). Commercially available potting soil was used for plant cultivation. During plant growth, the growth chamber (IBERCEX) temperature was maintained at 25:16 °C for day:night, with a photoperiod of 16:8-h light:dark and 70% relative humidity. During the experiment, uniform-sized plants were used after they had acclimated. To investigate the effects of polluted water (PW) on the biochemical composition of aquatic plants (macrophytes), a 2-week experiment was conducted in a batch system. The plants were exposed to 2 L of polluted water for either 7 or 14 days. After each exposure period, the plants’ responses and water conditions were analyzed. In the 7-day exposure group, the polluted water was replaced with fresh 2 L of polluted water after 7 days. However, in the 14-day exposure group, the same polluted water was maintained throughout the entire period, while any change in water level, due to evapotranspiration, was replenished using tap water. The water in the system remained static during the experiment. Changes in chlorophyll and carotenoid content, protein content, and catalase and ascorbate peroxidase activities were monitored. Furthermore, aerial biomass was also recorded. Each of the selected plants (approximately 2 months old, nearly uniform height) was subjected to the polluted groundwater sample (PW) at full strength. For controls, tap water was used (control conditions, CW). For each species, time, and treatment, a total of six independent biological replicates were used, with two experimental replicates in each case. Each tray was filled with 2 L of either polluted or tap water. Plant samples for biochemical analysis were collected after 7 and 14 days of exposure in the batch experiment.

#### Chlorophyll content of the macrophytes

For the extraction of the pigments of the different plant species, the plant samples (in triplicate for each treatment per plant) were homogenized with 8 mL of 80% acetone using a glass mortar and pestle. The resulting homogenate was centrifuged at 12,000 × *g* for 2 min (Lichtenthaler 1987). Subsequently, the supernatant was used to quantify the pigments spectrophotometrically. To calculate the total chlorophyll, chlorophyll a, chlorophyll b, and carotenoid contents (expressed in mg per gram of fresh weight), the following formulas were used:

1 $$\text{Chlorophyll A }\left(\text{Chla}\right)=\frac{\left(12.25 {\text{Abs}}_{663.2} -2.79 {\text{Abs}}_{646.8}\right)\times V }{1000 \times W}$$

2 $$\text{Chlorophyll B }\left(\text{Chlb}\right)=\frac{\left(21.50 {\text{Abs}}_{646.8} -5.10 {\text{Abs}}_{663.2}\right)\times V}{1000 \times W}$$

3 $$\text{Total chlorophyll }\left(\text{TChl}\right) = \text{Chla} + \text{Chlb}$$

4 $$\text{Total caroteniods }\left(\text{Car}\right)=\frac{\left(1000 {\text{Abs}}_{470} -1.82 \text{Chla}-85.02 \text{Chlb}\right)\times V}{198 \times 1000 \times W}$$

where Abs_663.2_ is the absorbance recorded at 663.2 nm, Abs_646.8_ is the absorbance recorded at 646.8 nm, Abs_470_ is the absorbance recorded at 470 nm, *V* is the volume in μL of the solution used for analysis in a spectrophotometer (Synergy HT BioTek, Vermont USA), and *W* is the weight of fresh biomass in grams used in the preparation.

### Plant enzyme activities and total soluble protein

The plant extracts (in triplicate for each treatment per plant) were prepared using 100 mg of leaf sample, 1 mL of extraction buffer (0.5 M NaH_2_PO_4_, 1 mM EDTA, pH 7.5, and 1% polyvinylpyrrolidone) (Li et al. 2013). The tissue was homogenized in a mortar and pestle under ice-cold conditions. The resulting homogenized sample was centrifuged at 13,300 rpm (~ 12,000 g) for 12 min at 4 °C. The supernatant was collected, and an aliquot of each sample (~ 50 µL) was saved for protein analysis. The total soluble protein content under cold working conditions was determined according to Bradford (1976). Specifically, Protein Assay Dye Reagent (Bio-Rad Laboratories Inc., USA) was used to determine the total protein in a crude extract of 10 µL well^−1^. Calibration curves from 0.1 to 0.5 µg µL^−1^ were prepared using bovine serum albumin (BSA) as a standard. The remaining extract was used to determine ascorbate peroxidase (APX) and catalase (CAT) activity. For catalase (CAT, EC 1.11.1.6) enzyme activity determination, 10 µL of plant extract and 186 µL of reaction buffer were mixed. The composition of the reaction buffer was 0.1 M NaH_2_PO_4_ and 1 mM EDTA, pH 7.5. For the blank, instead of adding plant extract, 10 µL of extraction buffer (0.1 M NaH_2_PO_4_, 1 mM EDTA, pH 7.5, 1 mM ascorbate) was added. After this, the absorbance was measured at 240 nm for 3 min to confirm the sterility of the mixture. Once the sterility of the mixture was confirmed, 4 µL of 2 M H_2_O_2_ was added to the respective wells. The change in absorbance was noted at a wavelength of 240 nm for 5 min (Nawaz et al. 2023). Catalase activity was expressed as mmol of H_2_O_2_ eliminated per minute per mg of protein using a molar extension coefficient (*ε*) of 39.4 mM^−1^ cm^−1^. Similarly, the ascorbate peroxidase (APX, EC 1.11.1.11) activity was quantified using 186 µL of reaction buffer (0.1 M NaH_2_PO_4_, 1 mM EDTA, pH 7.5) with 10 µL of leaf extract. A blank with 10 µL of extraction buffer instead of leaf extract was also prepared (Li et al. 2013). After confirming the sterility of the mixture at 290 nm (using the same method described for CAT activity), 2 µL of 20 mM H_2_O_2_ was added to the reaction mixture, and the changes in absorbance were noted for 5 min at a wavelength of 290 nm. For APX (*ε* = 2.8 mM^−1^ cm^−1^), the results are reported in µmol of oxidized ascorbate per minute per mg of protein.

### Mesocosm design and operational conditions

Based on the findings of the batch experiment, an HRT-based treatment system (15 and 30 days) was prepared. The HRT system consisted of multiple 19.5 × 19 × 19 cm polypropylene containers, with a total maximum volume of 4 L and was used for each treatment individually. The containers were filled to 3.5 L with medium–fine gravel (10–4.75 mm). The porosity (*Φ*) of the gravel used was 39%, the specific yield (*S*_*y*_) reached 33%, and the specific retention (*S*_*r*_) was 6%. Young sprouts of *P. australis*, *S. holoschoenus*, and *T. angustifolia* (three plants per treatment) were planted in each treatment. In summary, a total of 36 4-L mesocosms were prepared, which were composed of three different macrophytes exposed to two different HRTs (15 and 30 days) with two types of water (polluted and clean) in triplicate. The plants were acclimatized to clean water for 2 weeks. The different HRTs in the individual mesocosm were maintained using specified the flow rates. To determine the impact of hydraulic retention times (HRTs) of 15 and 30 days, flow rates (*Q*_*s*_*)* of 92- and 46-mL day^−1^, respectively, were used, as described by Ghosh and Gopal (2010). Considering the stated HRT (*t*), the porosity of the gravel used (*Φ*), the depth of the system (*y*), and the area (*A*). The flow rates were identified using the following equation.

5 $$\text{HRT} \left(t\right)=\frac{\text{Ay}\Phi }{Q}$$

The HRT becomes relevant in this setup because it represents the average time a specific amount of water spends within the system. The polluted or clean water was fed to the top of the system using a drip connected to flow regulators. The experiment was conducted for 4 weeks. Supplementary Fig. 1 shows images of the experimental setup and containers used for the treatments. The porosity of the gravel used in the system was 38.97%, while the specific yield from the system was 33.46%, and the specific retention was 5.51%. This indicates that among the total void space of 38.97%, upon filling the wetland system, a total of 5.51% of the water was retained on the gravel, while 33.46% was freely available. The system mimicked a vertical subsurface flow (VSSF) wetland, with approximately 1.37 L of water held within the void spaces of the gravel and an additional 0.5 L of water above the gravel layer, facilitating the development of a saturated zone crucial for VSSF function.

After the proposed incubation of the plants, the fresh and dry biomass of the roots and aerial parts of all the aquatic plants was quantified gravimetrically and is expressed in g ± g. For the fresh biomass, the samples were weighed immediately after harvesting; however, for the dried biomass, the plant tissues were weighed after drying at 60 °C (to a constant weight). With the help of fresh and dried biomass, the water storage capacity (g g^−1^) of the dried biomass of plant parts was calculated (Wu et al. 2021) using the following equation:

6 $$\text{Water storage capabilty}= \frac{\text{fresh weight}-\text{dried weight}}{\text{dried weight}}$$

#### Physical and chemical characterization of water

The polluted water used had a similar source, as mentioned earlier in section “Collection and characterization of polluted groundwater.” Water parameters, including pH, oxidative reduction potential (ORP), electrical conductance (EC), resistivity (RES), total dissolved solids (TDS), salinity (SAL), and dissolved oxygen (DO), were measured using a water quality multimeter (HI 98194 Multiparameter, Hanna Instruments, USA). The soluble orto-phosphate, ammonium, and nitrate were assessed using a segmented flow autoanalyzer (San ++ Skalar, Breda, The Netherlands) using the blue molybdate method, the indophenol blue method, and the Griess-Ilosvay reaction after reducing nitrate to nitrite in a copperized Cd column, respectively. To maintain the accuracy and precision of the analysis, standards ranging between 0.01 and 10.00 mg L^−1^ for P-PO_4_^−3^, N-NH_4_^+^, and N-NO_3_^−^ were introduced, and for quality assurance, an added drift of 5 mg L^−1^ and ultrapure water were added after every 15 samples were analyzed. The accuracy and precision of nutrient analyses using San ++ remained between 98 and 96%.

### Metal(loid) quantification in plants and water

Water samples were centrifuged, filtered, and acidified using concentrated HNO_3_ (1:9 v:v), whereas dried plant tissue samples were acid-digested. The respective samples were weighed (0.25 mg) and placed in Teflon microwave tubes, to which 2 mL of H_2_O_2_ (33%) and 8 mL of concentrated nitric acid (65%) were added. A standard (ERM-CD281 Rye grass-certified material) and blanks were also included. The sealed Teflon tubes were placed in a microwave digester (ETHOS ONE, Millestone, USA) at 190 °C for 20 min. The digestate was filtered (Scharlau CF/WASH110 filter paper, Ø 110 mm) and diluted with deionized water to a final volume of 25 mL. The metal(loid) contents were analyzed spectrophotometrically using ICP‒OES (SpectroGenesis or Arcos, AMETEC, Germany) and ICP-MS/MS (8900, Agilent, USA).

### Toxicity assessment using a *V. fischeri* bioluminescence inhibition assay

The impact of the HRT and plant type on metal(loid) phytoremediation can be assessed by studying the changes in the pattern of bioluminescence produced by *V. fischeri* (Rumbo et al. 2021). The bacterial suspension was prepared using individual highest luminant *V. fischeri* colonies. The suspension was subjected to centrifugation at 3600 × *g* for 15 min, and the resulting pelleted cells were resuspended in 5 mL of 2% NaCl (w/v) and maintained at 10 °C for 30 min. A 96-well opaque microplate containing 90 μL of water (polluted and clean, collected at the end of the experiment), a positive control (ZnSO_4_·7H_2_O, 219.8 mg L^−1^ in 2% NaCl) and a negative control (2% NaCl) was prepared. Later, 10 μL of the bacterial suspension was added to each well, and the changes in the luminescence were immediately measured (initial peak value) using a microplate reader (Synergy HT, BioTek, Vermont, USA). After initial peak value determination, the plate was incubated in a Thermomixer at 800 rpm and 15 °C, and the bioluminescence of *V. fischeri* was recorded at 5-min intervals for 30 min in a microplate reader.

### Statistical analysis

For each variable, the mean and standard deviation were computed using a minimum of three independent experimental repetitions. Kolmogorov‒Smirnov and Levene tests were employed to assess the normality and homogeneity of variances, respectively, with treatment serving as the fixed factor. The data were examined using one-way ANOVA, with a significance level of 0.05. The treatments were subsequently compared using Duncan’s post hoc tests. The Statistical Package for Social Sciences (SPSS v22.0 for Windows) and JASP (v0.17.1) were used for all the statistical analyses. The descriptive network analysis was performed using the Extended Bayesian Information Criterion Graphical Least Absolute Shrinkage and Selection Operator (EBICglasso) method, and a graphical network was constructed with the help of the Fruchterman-Reingold algorithm to construct the nodes (metals and metalloids) and edges (the interrelations).

## Results

### Plant biochemical profile after exposure to polluted water

**Fig. 1:**
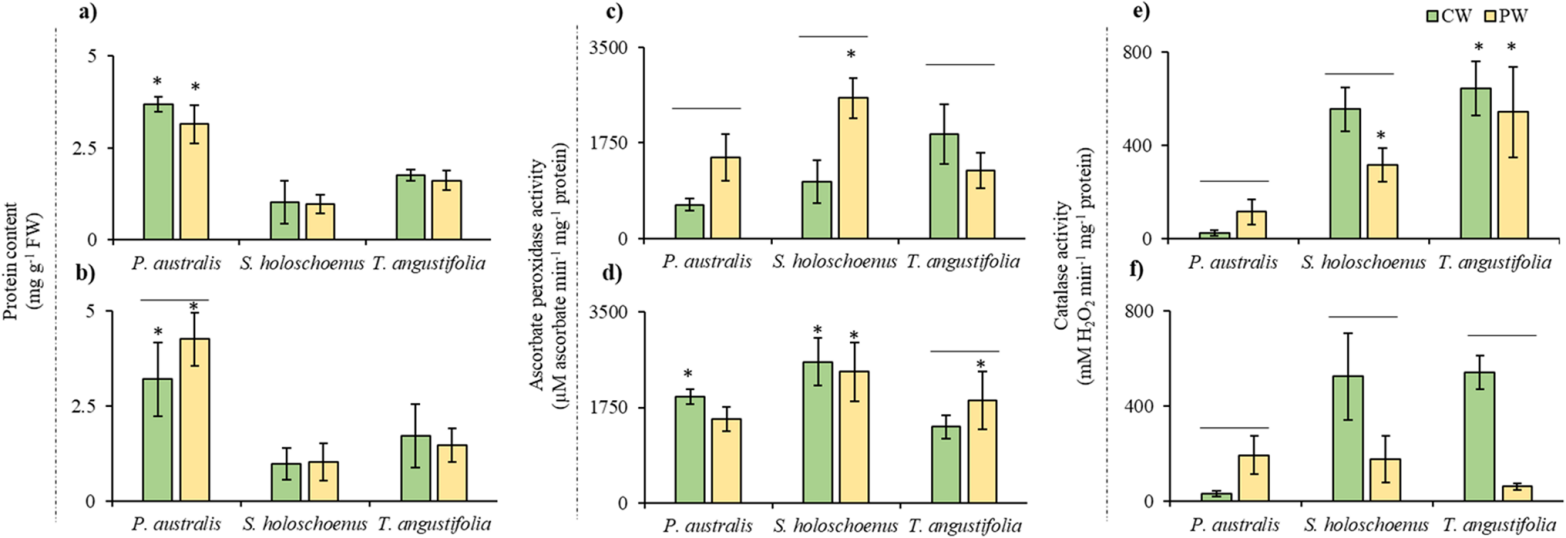
The biochemical profile of the studied aquatic plants, according to the duration of batch exposure (7 and 14 days, firsts, and seconds rows of pictures, respectively). Protein content in presented in (**a**) and (**b**), ascorbate peroxidase activity is presented in (**c**) and (**d**), and catalase activity is presented in (**e**) and (**f**). The star on each bar represents significant difference between the corresponding treatment at different HRT, while the line on bar represents significant difference between the different applied treatment at a specific HRT

The results of plant photosynthetic pigment production are presented in Table 1. In *Phragmites australis*, the concentration of total chlorophyll at 7 days was higher in plants (0.31 ± 0.14 mg g^−1^ FW) that grew in control water than in those that grew in contaminated water (0.20 ± 0.07 mg g^−1^ FW), as would be expected after stress. However, after 14 days, both concentrations were very similar. The chlorophyll ratio (Chl a/Chl b) at 7 days was higher in the PW treatment group, while it decreased at 14 days, indicating that after the onset of stress, the chlorophyll level decreased compared to that in the PW treatment group, while at 14 days, this decrease was not detected. Similarly, the TChl/Car ratio exhibited a similar pattern, indicating that the content of carotenoids, which are antioxidant pigments, increased at the beginning of exposure. After 7 and 14 days, *Scirpus holoschoenus* presented similar concentrations of total chlorophyll in the control and treated samples. The Chl a/Chl b and TChl/Car ratios are practically stable. The chlorophyll concentration (0.24 ± 0.09 mg g^−1^ FW) of *T*. *angustifolia* was notably greater at 14 days than in the control, and the TChl/Car ratio increased in contaminated samples after 14 days, indicating a possible protective role of carotenoids in this species. A general trend was observed that indicated that all the studied plants performed relatively well with exposure to the polluted water compared with the pigment production in the clean condition. However, with respect to the exposure time, the plants were more stable at 14 days, indicating that the performance of plants can be better for phytoremediation of metal(loid)s at higher HRTs, while acute and frequent exposure to metal(loid)s can impact plant growth, as seen in the 7-day exposure group. The concentration of total soluble proteins in *Phragmites australis* fluctuated at 7 and 14 days in the control samples (Fig. 1a and b). In the contaminated samples, the concentration increased notably at 14 days in plants cultivated in the PW treatment (4.25 ± 0.96 mg g^−1^ FW) compared to those cultivated in the CW treatment (3.19 ± 0.19 mg g^−1^ FW). The concentration of total soluble proteins in the PW of *Scirpus holoschoenus* was stable after 7 days and 14 days of batch exposure (BE) compared to that in the control group cultivated in clean water. Similarly, *Typha angustifolia* showed similar values after 7 and 14 days of exposure to stress.

**Table 1:** Photosynthetic pigment production by aquatic plants under metal(loid)s polluted water exposure at 7- and 14-day batch exposure time

Studied parameters	Days	*Phragmites australis*				*Scirpus holoschoenus*					*Typha angustifolia*			
		CW^1^		PW^1^		CW		PW			CW		PW	
Total Chlorophyll (TChl, mg g^−1^ FW^*^)	7	0.31 ± 0.14	aA	0.20 ± 0.07	bB	0.09 ± 0.01	aA	0.10 ± 0.06	aA	0.11 ± 0.03		aA	0.10 ± 0.02	aA
	14	0.31 ± 0.09	aA	0.29 ± 0.09	aA	0.13 ± 0.02	aA	0.14 ± 0.06	aA	0.15 ± 0.03		aA	0.24 ± 0.09	aA
Chlorophyll a (Chla, mg g^−1^ FW)	7	0.24 ± 0.11	aA	0.15 ± 0.03	bB	0.07 ± 0.01	aA	0.08 ± 0.01	aA	0.09 ± 0.02		aA	0.08 ± 0.00	aA
	14	0.25 ± 0.07	aA	0.23 ± 0.07	aA	0.10 ± 0.01	aA	0.11 ± 0.05	aA	0.12 ± 0.03		bB	0.19 ± 0.07	aA
Chlorophyll b (Chl b, mg g^−1^ FW)	7	0.06 ± 0.03	aA	0.04 ± 0.01	bA	0.02 ± 0.00	aA	0.02 ± 0.00	aA	0.02 ± 0.01		aA	0.02 ± 0.00	aA
	14	0.06 ± 0.02	aA	0.06 ± 0.02	aA	0.03 ± 0.00	aA	0.03 ± 0.01	aA	0.04 ± 0.01		aA	0.05 ± 0.02	aA
Carotenoids (Car, mg g^−1^ FW)	7	0.11 ± 0.06	aA	0.08 ± 0.01	bA	0.05 ± 0.00	aA	0.05 ± 0.01	aA	0.05 ± 0.01		aA	0.05 ± 0.00	aA
	14	0.12 ± 0.03	aA	0.11 ± 0.02	aA	0.06 ± 0.01	aA	0.07 ± 0.02	aA	0.07 ± 0.01		aA	0.08 ± 0.03	aA
Chl a/Chl b	7	3.90 ± 0.15	aA	3.99 ± 0.05	aA	3.48 ± 0.09	aA	3.61 ± 0.10	aA	3.66 ± 0.17		aA	3.67 ± 0.14	aA
	14	3.86 ± 0.07	aA	3.61 ± 0.12	bA	3.24 ± 0.07	aA	3.43 ± 0.14	aA	3.36 ± 0.07		aA	3.61 ± 0.13	aA
TChl/Car	7	2.66 ± 0.12	aA	2.57 ± 0.08	aA	1.87 ± 0.08	aA	1.87 ± 0.04	aA	2.11 ± 0.21		aA	1.95 ± 0.06	bA
	14	2.69 ± 0.06	aA	2.68 ± 0.55	aA	2.02 ± 0.05	aA	2.05 ± 0.21	aA	2.22 ± 0.17		aB	2.89 ± 0.29	aA

^1^CW meant clean water, while PW meant polluted water. ^*^FW fresh weight. The post hoc test analysis between CW and PW for specific parameters in a plant type at different HRTs is presented by alphabet. “a” alphabet in small fonts showing significantly highest value followed by later alphabets, while “A” alphabet in large fonts represent significant difference between treatments in which plant were cultivated at a specific HRT

The ascorbate peroxidase activity of the plants cultivated at different hydraulic retention times is presented in Fig. 1c and d for 7 and 14 days of BE, respectively. *Phragmites australis* and *Scirpus holoschoenus* exhibited similar APX activity (µM ascorbate min^−1^ mg^−1^ protein). With 7 days of BE, the plants cultivated on polluted water (PW) showed greater activity (1490.79 ± 425.16 and 2570.38 ± 369.22 for *P. australis* and *S. holoschoenus*, respectively), which was stable at 14 days of BE, and *Typha angustifolia* showed increased ascorbate peroxidase activity in the plants cultivated with polluted water at 14 days of BE compared to the control. The control samples showed decreased activity at a BE of 7 days; however, at 14 days of BE, the APX activity increased noticeably for *P. australis* and *S. holoschoenus*.

The catalase activity (mM H_2_O_2_ min^−1^ mg^−1^ protein) in the control treatment for *P. australis* was very low (24.67 ± 11.11) at 7 day of BE, while plants cultivated in polluted water had higher levels of CAT (115.53 ± 52.69). With 14 days of BE, the activity in *P. australis* remained similar, with plants in the PW showing higher CAT activity than plants in clean water. *Scirpus holoschoenus* presented lower CAT activity in the PW treatment and a slight decrease after 14 days. In *T. angustifolia*, this activity decreased considerably after 14 days and was lower in PW-exposed plants than in control plants. In the three species, with 14 days of BE, the catalase activity was lower in the contaminated samples than in the control samples.

### Physiological response of the plants used in the mesocosm experiment

Based on the findings of the biochemical response of the selected macrophyte for the phytoremediation of polluted groundwater, a mesocosm experiment was conducted. The canopy cover of *P. australis* and *S. holoschoenus* was much broader and denser than that of *T. angustifolia* during the experiment (Supplementary Fig. 1). Similar results were also evident for the biomass produced by the plants (Table 2). This indicated that the plants had variable responses to the provided conditions, i.e., different HRTs. At the time of harvest, the shoot biomass production status of the *P. angustifolia* plants showed no change in the aerial phenotype or in the shoot fresh biomass produced with 30 days of HRT; however, a slight decrease was noted at 15-day HRTs (~ 5 to 8% for fresh and dry biomass of shoot and root). The water storage capacity (WSC, g g^−1^ DW) was higher for the shoots in the control treatment, while this parameter was greater for the roots in the treatment with polluted water (ranging from 2.37 ± 0.22 and 2.38 ± 0.22 at the 30- and 15-day HRTs, respectively). The fresh biomass for roots and shoots and the WSC for *S. holoschoenus* were significantly higher at both HRTs, with higher biomass noted at 15 days of HRT, which indicated higher plant potential to withhold metal(loid)s in groundwater with less impact on growth. A negative impact of the higher dosing rate (15 days of HRT) was found for *T. angustifolia*, which showed less fresh biomass production for both shoots (up to 35% less) and roots (up to 10% less) compared to the control at both HRTs but showed greater dry biomass.

**Table 2:** Biomass production and water storage capacity of aquatic plants under metal(loid)s polluted water exposure at different exposure HRTs

Studied parameter		HRT	*Phragmites australis*				*Scirpus holoschoenus*				*Typha angustifolia*			
			CW^1^		PW^1^		CW		PW		CW		PW	
Shoot	FW^2^	30	9.50 ± 0.15	aB	9.94 ± 0.28	aA	10.13 ± 0.76	aB	24.20 ± 2.59	aA	11.21 ± 0.10	aA	7.22 ± 0.48	aB
	(g)	15	9.38 ± 0.29	aA	9.54 ± 0.57	bA	10.01 ± 0.75	aB	26.32 ± 2.49	bA	11.07 ± 0.19	aA	6.94 ± 0.96	bB
	DW^2^	30	1.18 ± 0.14	aB	1.40 ± 0.07	aA	2.40 ± 0.05	aA	2.07 ± 0.02	aB	0.52 ± 0.05	aB	2.76 ± 0.10	aA
	(g)	15	1.05 ± 0.16	aA	1.17 ± 0.08	bA	2.14 ± 0.15	aA	2.26 ± 0.02	bA	0.46 ± 0.05	aB	2.32 ± 0.11	bA
	WSC^2^	30	7.02 ± 0.86	aA	6.12 ± 0.16	aB	3.22 ± 0.22	aB	10.68 ± 1.13	aA	20.58 ± 1.82	aA	1.62 ± 0.08	aB
	(g g^−1^ DW)	15	7.91 ± 1.06	aA	7.13 ± 0.06	bB	3.69 ± 0.25	aB	10.65 ± 1.01	bA	22.94 ± 2.33	aA	1.99 ± 0.28	bB
Root	FW^2^	30	3.12 ± 0.34	aA	2.90 ± 0.35	aA	5.18 ± 0.47	aB	9.44 ± 0.94	aA	1.99 ± 0.18	aB	2.26 ± 0.38	aA
	(g)	15	3.36 ± 0.36	aA	2.54 ± 0.37	bB	5.58 ± 0.50	bB	10.16 ± 0.82	bA	2.14 ± 0.19	bA	1.99 ± 0.41	bB
	DW^2^	30	1.18 ± 0.14	aA	0.86 ± 0.05	aB	2.40 ± 0.17	aB	3.18 ± 0.14	aA	0.52 ± 0.05	aA	0.63 ± 0.04	aA
	(g)	15	1.17 ± 0.17	aA	0.75 ± 0.05	bB	2.37 ± 0.17	aB	3.77 ± 0.11	bA	0.51 ± 0.06	aA	0.56 ± 0.05	bA
	WSC^2^	30	1.64 ± 0.03	aB	2.37 ± 0.22	aA	1.16 ± 0.04	aB	1.97 ± 0.17	aA	2.82 ± 0.01	aA	2.57 ± 0.37	aA
	(g g^−1^ DW)	15	1.87 ± 0.11	aB	2.38 ± 0.25	aA	0.05 ± 2.00	aB	1.69 ± 0.14	aA	3.17 ± 0.09	aA	2.57 ± 0.43	aA

^1^CW is clean water, while PW is polluted water, ^*2*^*FW* fresh weight, *DW* dried weight, and *WSC* water storage capacity. The post hoc test analysis is presented by “a” alphabet in small fonts between different HRTs showing significantly highest value followed by later alphabets, while “A” alphabet in large fonts represent significant difference between treatments in which plant were cultivate at a specific HRT

### Physical and chemical parameters of groundwater under different HRT treatment regimes

**Fig. 2:**
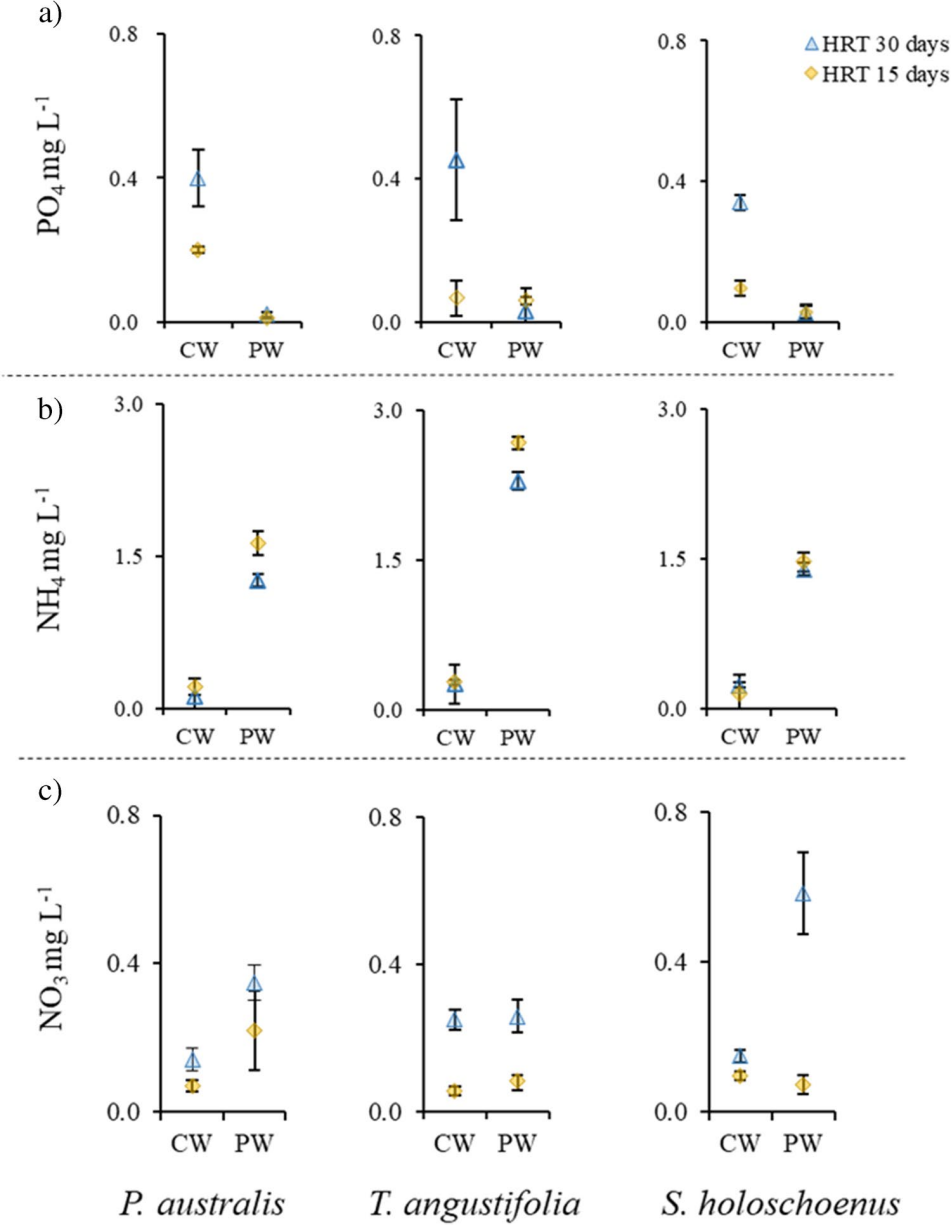
Levels of nutrients (mg L^−1^) in the water for control and polluted water treatments, cultivated with aquatic plants at studied HRT (15 and 30 days). **a** PO_4_^−3^, **b** NH_4_^+^, and **c** NO_3_^−^, the yellow diamonds “♦” showing the values of respective parameter at 15-day HRT, while blue triangles “▲” showing the values at 30-days HRT

**Fig. 3:**
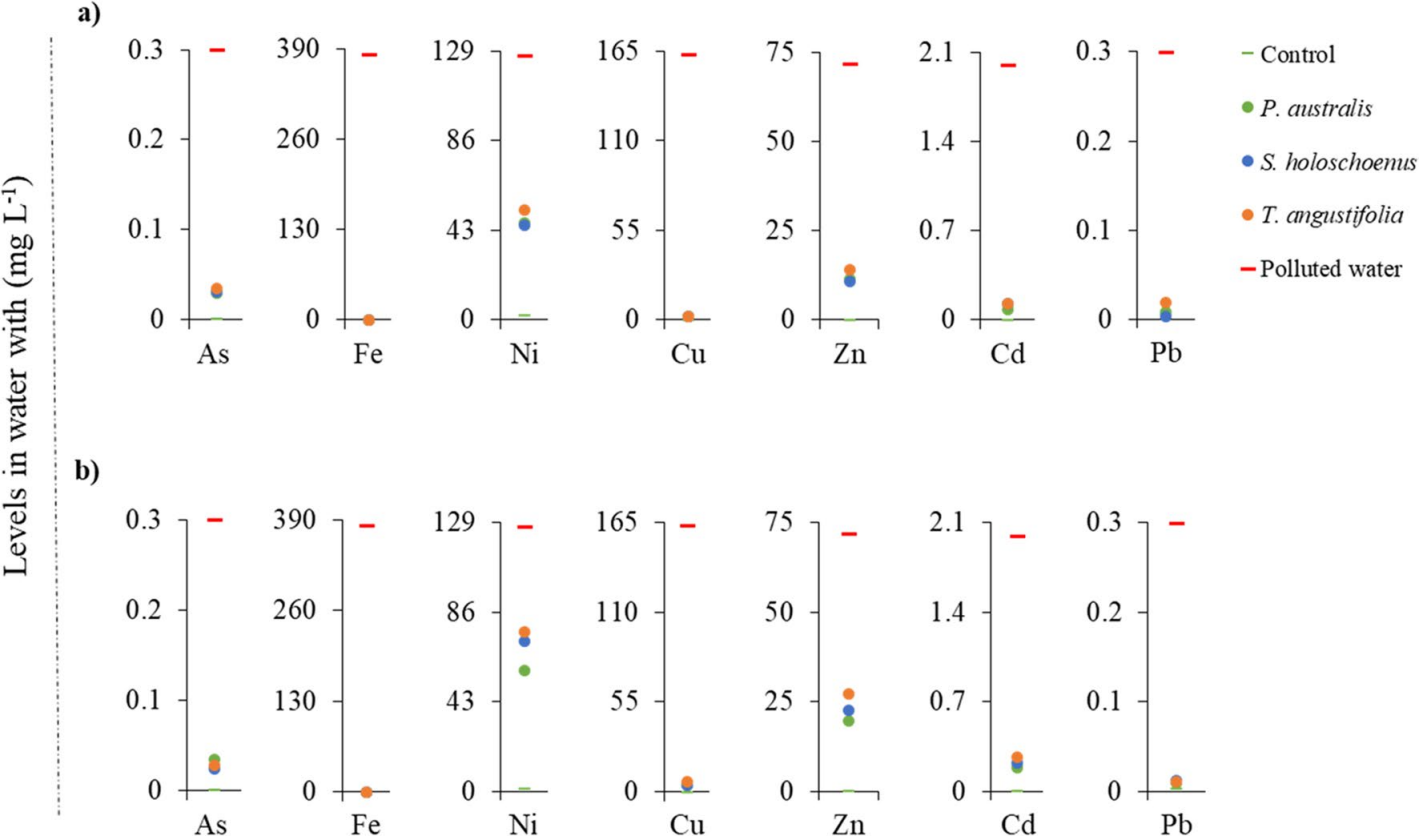
The metal and metalloids content in polluted water after phytoremediation by aquatic plants. Panel **a** indicates metal(loid)s content in water with HRT of 30 days, while panel **b** indicates the metal(loid)s content in water with HRT of 15 days

**Fig. 4:**
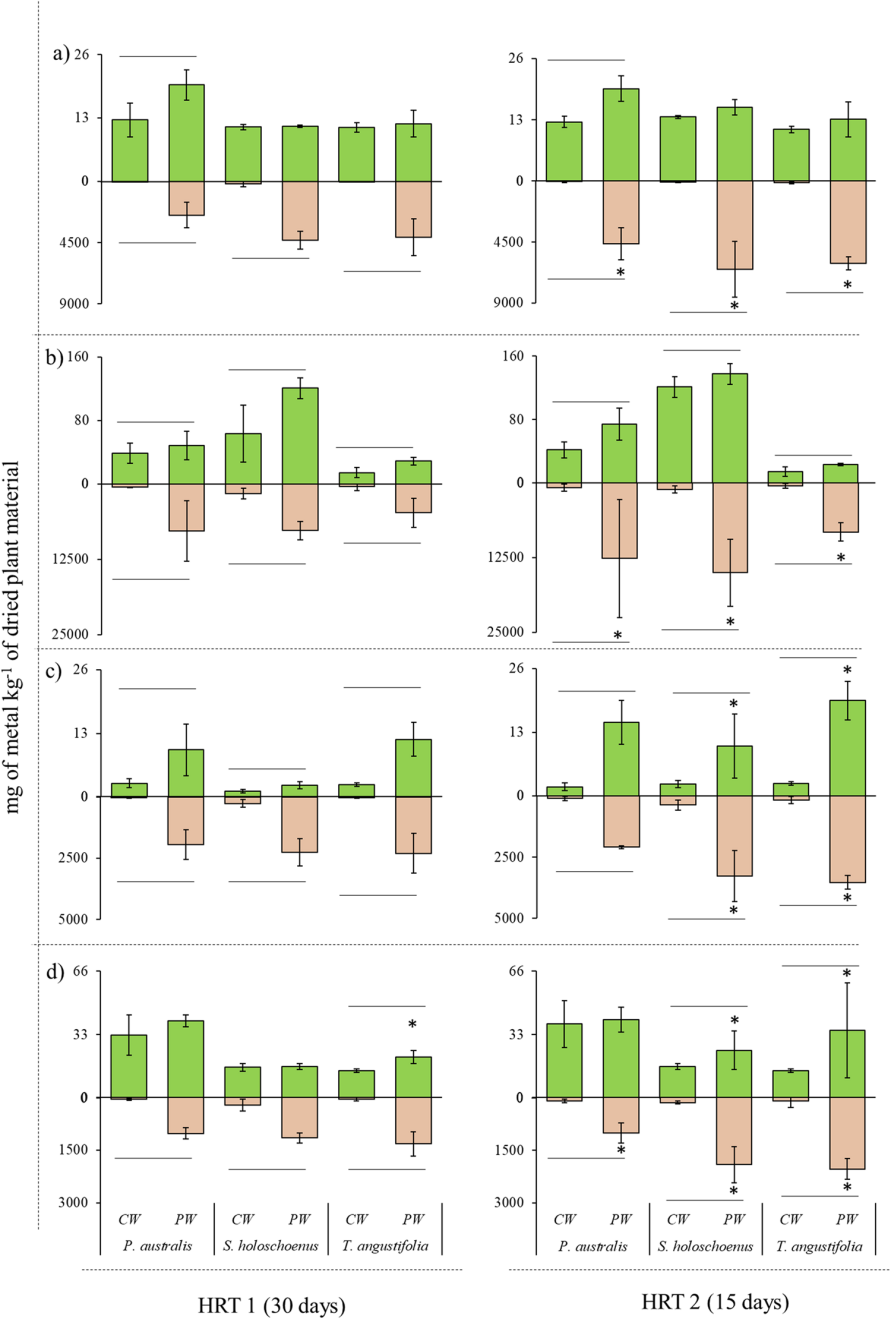
Essential metals uptake, with **a** Cu, **b** Fe, **c** Ni, and **d** Zn, in plant with reference to different HRTs. The asterisk (*) on the bar indicates significantly higher level detected between the specific treatment (CW or PW) within the plant part (root or shoot). While lines between bars of CW and PW indicates significant difference detected in plant part (root or shoot) for a specific plant

**Fig. 5:**
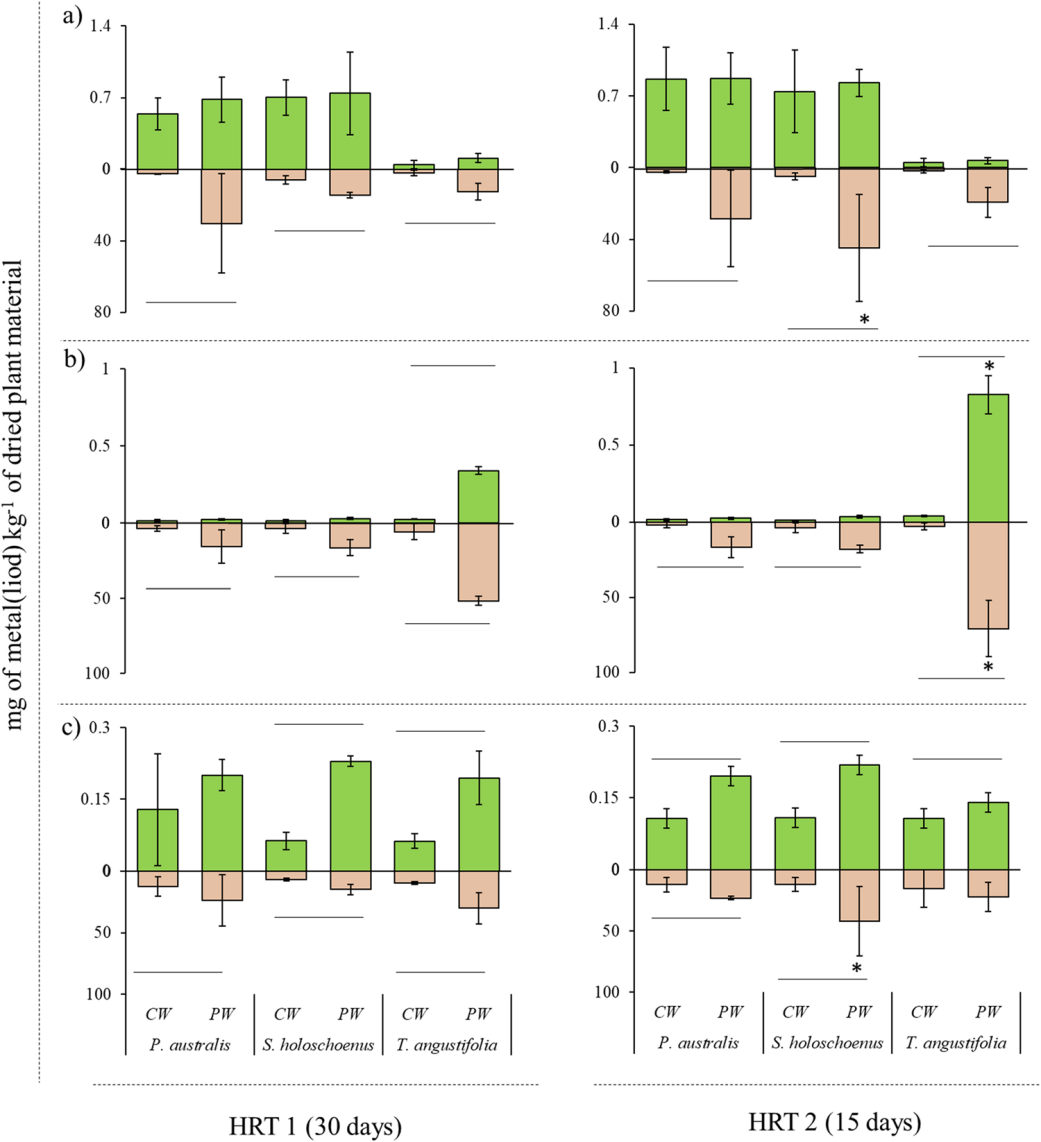
Nonessential metal(loid)s uptake, with **a** As, **b** Cd, and **c** Pb, in plant with reference to different HRTs. The asterisk (*) on the bar indicates significantly higher level detected between the specific treatment (CW or PW) within the plant part (root or shoot). While lines between bars of CW and PW indicates significant difference detected in plant part (root or shoot) for specific plant

The studied physical and chemical parameters of the control and polluted water, with the cultivation of macrophytes at different HRTs, are presented in Table 3. The parameters were noted at intervals of 15 days and 30 days for both hydraulic retention regimes. All the physical and chemical parameters of CW at both HRTs at both monitoring times were found to be significantly more stable than those of PW. The initial pH of the polluted groundwater was 2.7. The pH with PW at the 15-day HRT decreased with increasing exposure duration, while at the 30-day HRT, the pH remained relatively close to that of CW for all plants, ranging from 7.21 ± 0.07 for *P. australis* to 7.49 ± 0.12 for *S. holoschoenus.* This indicated that high retention of polluted water in mesocosms provides enough time for plants to buffer the pH, while with lower HRTs, a gradual decrease in the pH can impact the water phytoremediation capacity of plants. In the present study, this can be due to changes in water pH, as these metals are highly mobile under acidic conditions, while upon contact with the growing substrate (gravel), the pH of PW became more neutral (Table 2) or due to phytostabilization of essential nutrients and metals, along with subsequent uptake by plants (Figs. 2, 3, 4, and 5). A longer exposure duration increased the EC, TDS, and salinity. This can be due to the production of root exudates by plants, which are known to influence these parameters (Padmavathiamma and Li 2012; Elmajdoub et al. 2014). The resistivity of water decreased in the present study with increasing exposure duration at both HRTs. This is a clear indication that plant rhizospheric activities increase metal and metal(loid) removal due to adsorption, absorption, and precipitation, contributing to reduced water resistivity, as it is reciprocal to the increase in EC, salinity, and TDS (Rahman et al. 2022). The ORP for PW was significantly greater than that for CW in all the plants. A higher positive ORP is an indication of the presence of oxidizing agents in the water, which can lead to plant stress (Ma et al. 2016); however, in the present study, the ORP remained statically like that in the CW treatment, especially at 30 days of HRT on the 30th day of exposure (except for *T. angustifolia*), showing that the plants were tolerant to the provided conditions. An increase in the EC is an indication of a higher presence of salts in water, which is evident in the present study.

**Table 3:** Physical and chemical parameters of water monitored during the phytoremediation at different HRTs using aquatic macrophytes

Studied parameter	HRT	ED*	*Phragmites australis*				*Scirpus holoschoenus*				*Typha angustifolia*			
			CW^1^		PW^1^		CW		PW		CW		PW	
pH	15	15	7.88 ± 0.06	aA	7.24 ± 0.02	aB	7.54 ± 0.39	aA	7.14 ± 0.06	aB	7.83 ± 0.10	aA	7.32 ± 0.18	bB
		30	7.68 ± 0.15	aA	7.08 ± 0.05	bB	7.68 ± 0.15	aA	7.05 ± 0.04	bB	8.04 ± 0.05	aA	7.53 ± 0.11	aB
	30	15	7.86 ± 0.08	aA	7.08 ± 0.11	bB	7.89 ± 0.02	aA	7.26 ± 0.05	aB	7.88 ± 0.15	aA	7.15 ± 0.05	aB
		30	7.59 ± 0.10	bA	7.21 ± 0.07	aB	7.82 ± 0.11	aA	7.49 ± 0.12	aB	7.90 ± 0.04	aA	7.32 ± 0.25	bB
ORP [V]	15	15	0.15 ± 0.00	aB	0.25 ± 0.00	aA	0.16 ± 0.01	aB	0.25 ± 0.00	aA	0.14 ± 0.00	aB	0.25 ± 0.01	aA
		30	0.15 ± 0.00	aB	0.25 ± 0.00	aA	0.15 ± 0.00	aB	0.25 ± 0.00	aA	0.15 ± 0.00	aB	0.24 ± 0.01	aA
	30	15	0.15 ± 0.00	aB	0.24 ± 0.00	aA	0.15 ± 0.00	bB	0.25 ± 0.00	aA	0.15 ± 0.00	aB	0.24 ± 0.01	aA
		30	0.23 ± 0.00	bB	0.26 ± 0.00	aA	0.24 ± 0.01	aA	0.25 ± 0.01	aA	0.22 ± 0.00	aB	0.27 ± 0.00	aA
EC [dS m^−1^]	15	15	2.39 ± 1.00	aB	20.07 ± 7.99	aA	2.35 ± 0.91	aB	18.93 ± 7.58	aA	0.98 ± 0.37	aB	20.74 ± 7.88	aA
		30	2.43 ± 0.83	aB	21.24 ± 7.91	aA	1.88 ± 0.74	bB	21.79 ± 8.29	aA	1.20 ± 0.46	aB	21.12 ± 8.01	aA
	30	15	2.43 ± 0.83	aB	21.59 ± 8.61	bA	1.88 ± 0.74	aB	21.79 ± 8.29	bA	1.20 ± 0.46	bB	21.12 ± 8.01	bA
		30	2.68 ± 0.71	bB	31.69 ± 1.08	aA	2.14 ± 0.39	bB	31.23 ± 0.64	aA	1.50 ± 0.05	aB	31.62 ± 1.39	aA
TDS [ppt]	15	15	0.12 ± 0.05	aB	1.00 ± 0.40	bA	0.12 ± 0.05	aB	0.95 ± 0.38	bA	0.05 ± 0.02	aB	1.04 ± 0.39	bA
		30	0.16 ± 0.03	aB	1.57 ± 0.02	aA	0.14 ± 0.03	aB	1.33 ± 0.07	aA	0.07 ± 0.00	aB	1.50 ± 0.02	aA
	30	15	0.12 ± 0.04	aB	1.08 ± 0.43	bA	0.09 ± 0.04	aB	1.09 ± 0.41	bA	0.06 ± 0.02	aB	1.06 ± 0.40	bA
		30	0.13 ± 0.04	aB	1.58 ± 0.05	aA	0.11 ± 0.02	aB	1.56 ± 0.03	aA	0.08 ± 0.00	aB	1.58 ± 0.07	aA
Sal [psu]	15	15	0.11 ± 0.05	aB	1.03 ± 0.43	aA	0.11 ± 0.04	aB	0.97 ± 0.41	bA	0.05 ± 0.02	aB	1.07 ± 0.43	bA
		30	0.15 ± 0.03	aB	1.65 ± 0.03	aA	0.13 ± 0.03	aB	1.38 ± 0.08	aA	0.07 ± 0.00	aB	1.56 ± 0.02	aA
	30	15	0.12 ± 0.04	aB	1.11 ± 0.47	aA	0.09 ± 0.04	aB	1.12 ± 0.45	bA	0.06 ± 0.02	aB	1.09 ± 0.44	bA
		30	0.13 ± 0.03	aB	1.66 ± 0.06	aA	0.10 ± 0.02	aB	1.63 ± 0.04	aA	0.07 ± 0.00	aB	1.66 ± 0.08	aA
RES [Ohm m]	15	15	4.91 ± 2.43	aA	0.54 ± 0.16	aB	4.65 ± 1.37	aA	0.58 ± 0.17	aB	11.27 ± 3.75	aA	0.52 ± 0.15	aB
		30	3.33 ± 0.77	bA	0.32 ± 0.00	bB	3.84 ± 0.97	bA	0.38 ± 0.02	bB	7.14 ± 0.36	bA	0.33 ± 0.00	bB
	30	15	4.58 ± 1.56	aA	0.51 ± 0.15	aB	5.91 ± 2.04	aA	0.50 ± 0.14	bB	9.13 ± 2.67	aA	0.51 ± 0.14	aB
		30	3.91 ± 0.94	bA	0.32 ± 0.01	bB	4.80 ± 0.82	bA	0.32 ± 0.01	aB	6.67 ± 0.23	bA	0.32 ± 0.01	bB

^*^ED meant exposure duration (15–30 days), while HRT is hydraulic retention time in the mesocosm, ^1^CW is clean water, while PW is polluted water, the post hoc test analysis is presented by “a” alphabet in small fonts between different HRTs showing significantly highest value followed by later alphabets, while “A” alphabet in large fonts represent significant difference between treatments in which plant were cultivate at a specific HRT

The levels of PO_4_^−3^, NH_4_^+^, and NO_3_^−^ (mg L^−1^) in the CW and PW at applied HRTs (15 and 30 days) are presented in Fig. 2. The PO_4_^−3^ content (mg L^−1^) in the tap water used for the experiment was ~ 0.06, while that in the polluted water was ~ 0.32. The level of PO_4_^−3^ in the controls at the 30-day HRT was relatively greater than that at the 15-day HRT. This could be due to the microbial activity resulting in phosphate solubilization from the rooting substrate material in the rhizosphere (Wang et al. 2021), while with PW, no changes between the HRTs were noted in any of the plants, which indicates the precipitation of phosphate and metals in the form of insoluble metal phosphates, reducing the bioavailability of metals and phosphate to the plants. The initial level of NH_4_^+^ (mg L^−1^) in the tab water used for the experiment was ~ 0.03, while that in the polluted water was ~ 6.8. The impact of HRT on macrophyte phytoremediation was also evident in the treated groundwater. Except in the case of *T. angustifolia*, significant changes were noted in the levels of ammonia with respect to different HRTs. With respect to phytoremediation, a significant decrease in NH_4_^+^ was noted with both HRTs; however, for *P. australis* and *S. holoschoenus*, significantly higher removal was noted at an HRT of 30 days. This showed that the plants studied in the present investigation could efficiently assist in the removal of ammonia from PW. The process of microbial-assisted nitrification in the rhizosphere of plants results in the oxidization of ammonia to nitrite and then nitrate (Robles-Porchas et al. 2020). This surely resulted in higher level of nitrate with improved nitrification, which was also apparent in the pattern of NO_3_^−^ content in the treated water in the present study (Fig. 2c), indicating that higher nitrate levels were present at the 30-day HRT for all plants in both CW and PW. The initial nitrate concentration (mg L^−1^) in the tab water was ~ 0.01, while that in the polluted groundwater used in the present study was ~ 0.41. Nitrate in water is highly soluble because it can cause eutrophication in aquatic systems, and hence is considered a ubiquitous pollutant (Liu et al. 2022). Nitrate levels increase from the initial concentration; however, nitrates are essential for plant growth (Wang et al. 2020); hence, they can be removed by cultivating macrophytes (Yao et al. 2019).

The profile of metal(loid)s in water after phytoremediation using aquatic plants with an HRT of 30 is presented in Fig. 3a, while that after 15 days of HRT treatment is presented in Fig. 3b. Higher retention times clearly indicated lower levels of metals and metalloids in the water. The highest removal was noted for Fe and Cu at both HRTs, for which the removal reached 90 to 95%. No significant differences in plant-dependent removal were found. The level of Ni in the water varied with respect to the plant type and HRT. Relatively greater decreases were detected at the 30-day HRT; however, *P. australis* and *S. holoschoenus* (Ni levels in water of 46.63 ± 6.34 and 45.16 ± 7.79 mg L^−1^, respectively) exhibited slightly greater decreases (~ 10%) than did *T. angustifolia* (52.88 ± 4.12 mg L^−1^). Despite the considerable reduction in the metal(loid) content with phytoremediation, the levels of As, Cd, and Pb remained within the permissible limits of the groundwater sanitation standards of Flanders. In addition, there was a slight increase in available Fe, Ni, and Pb in the control group, which may have been due to the weathering of gravel; hence, care should be taken when choosing a support medium for aquatic plants. A medium or substrate that contains high levels of metal(loid)s can become bioavailable with longer durations of exposure to the substrate.

### Metal(loid) uptake by aquatic plants at different HRTs

The response of plant metal and metalloid uptake and compartmentalization in the plant body is presented in Fig. 4 for essential metals and in Fig. 5 for nonessential metal(loid)s. Regarding metal and metal(loid) uptake and compartmentalization, significant differences were observed between the treatments and applied HRTs, which produced diverse HM uptake profiles with each treatment. The levels of essential metals were significantly greater in roots than in shoots, indicating that the plants used in the present study were more suitable for phytostabilization of Cu, Fe, Ni, and Zn. Furthermore, the uptake and translocation of essential metals were dose- and exposure-dependent, as higher levels of these essential metals were extracted and translated with a 15-day HRT, which resulted in significantly greater uptake of all essential metals. This was because more metals were available due to the shorter retention time. With an HRT of 30 days, the roots showed increases of up to 230 × for Cu, 87 × for Fe, 174 × for Ni, 35 × for Zn, 49 × for As, 149 × for Cd, and 199 × for Pb compared to the shoots. The control water also showed uptake of these metals, but the origin of these metals was likely weathering of the growth substrate and gravel. Similar results were noted for the nonessential metals As, Cd, and Pb, where at longer retention times (i.e., 30 days of HRT), plant uptake was significantly lower than that at 15 days of HRT, and further higher levels were also noted in roots than in shoots (Fig. 5). With an HRT of 15 days, the roots were 345 × for Cu, 155 × for Fe, 191 × for Ni, 44 × for Zn, 56 × for As, 99 × for Cd, and 332 × for Pb.

**Fig. 6:**
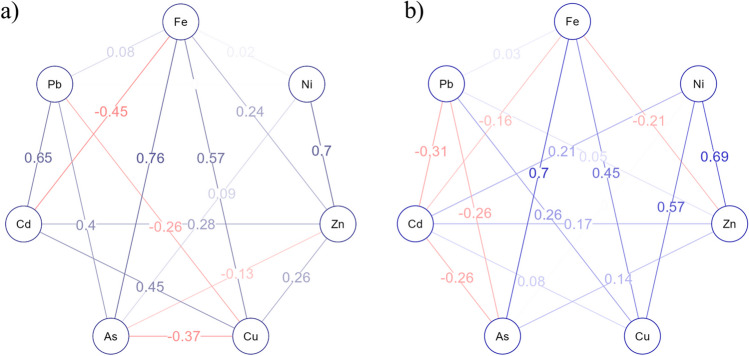
Descriptive network analysis using EBICglasso estimator based on Fruchterman-Reingold algorithm metal(loid)s content in plant. **a** Network constructed for the metal uptake by plants at HRT of 30 days, **b** network constructed for the metal uptake by plants at HRT of 15 days

The HRT is also known to affect metal uptake and removal by plants (Gaballah et al. 2021). To understand the impact of HRT on the interactions between the studied metals and metalloids in the macrophytes, a descriptive network analysis was performed using the EBICglasso estimator based on the Fruchterman-Reingold algorithm, and the results are presented in Fig. 6 (6.a for a 30-day HRT and 6.b for a 15-day HRT). The descriptive network graphs showed 17 significant edges (out of a total of 21 possible edges/links), with a sparsity of 0.190, indicating that the network construct was dense for both HRTs (Supplementary Table 1). The network graphs were constructed using the weight matrix score (Supplementary Table 2 for 30 days of HRT and Supplementary Table 3 for 14 days) and showed the nodes, i.e., metal(loid)s in plants (As, Cd, Cu, Fe, Ni, Pb, and Zn), in a circular layout connected with the edges or links between the nodes, indicating significant correlations, which are colored blue for positive or red for negative correlation, while the intensity of color indicates the strength or weakness of the correlation between the nodes. It is clear that at relatively high HRTs, plants accumulate relatively high levels of Cd, As, and Pb via synergistic uptake; however, the uptake of Fe negatively impacts the uptake of Cd (− 0.45), and Cu antagonizes As (− 0.45) and Pb (− 0.26) uptake in plants. In addition, As was also found to have an antagonistic relationship with Zn (− 0.13) at the 30-day HRT. With an HRT of 15 days, the uptake profile of essential metals seemed to interact more synergistically, while nonessential metal(loid)s interacted antagonistically. For instance, nonessential metal(loid)s, including As, Cd, and Pb, showed a significant negative correlation with plant uptake, indicating an antagonistic relationship with plant uptake, with the exception of Fe uptake, which antagonistically interacted with Zn (− 0.21) and Cd (− 0.16).

### Toxicological profiling of water with and without phytoremediation

**Fig. 7:**
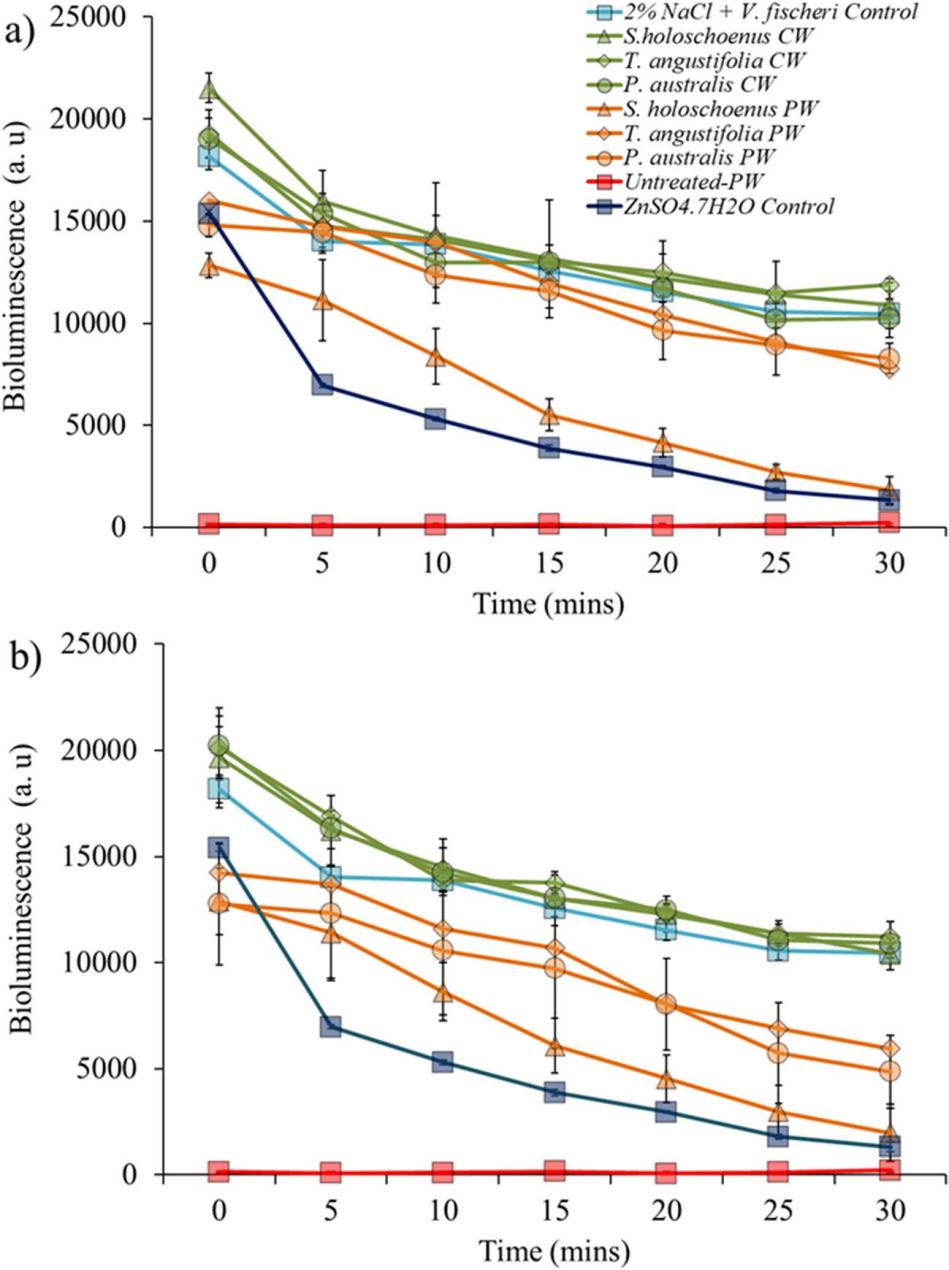
Bioluminescence assay for the studied HRT (15 and 30 days) with different selected plants with phytoremediation using aquatic plants, **a** bioluminescence assay conducted with 30-days HRT treatment, while **b** with 15-day HRT

The impact of HRT on the macrophyte-assisted phytoremediation of polluted groundwater was assessed using the bioluminescent bacteria *Aliivibrio fischeri*, also known as *Vibrio fischeri*. The results showed the variability in the toxicity displayed by *V. fischeri* with respect to the type of plant used and the HRT applied (Fig. 7). The bioluminescence emitted by the bacteria was observed over a 30-min duration at 5-min intervals to track its evolution. The bioluminescence of *V. fischeri* decreased almost entirely at the start of the experiment as soon as the bacteria encountered the polluted groundwater. For phytoremediation with all plants, an improvement in bioluminescence compared to that in polluted groundwater was observed (Fig. 6a and b). However, with an HRT of 15 days, the bioluminescence was lower than that of the controls, indicating that the water was still toxic (Fig. 7b). At an HRT of 15 days, the reduction in bioluminescence was 6 to 10% lower than that at an HRT of 30 days. This indicates that for effective macrophyte-assisted phytoremediation of PW, a longer retention time should be adopted to facilitate not only the removal of pollutants but also the stabilization of physical and chemical parameters that can reduce the toxicity of PW.

## Discussion

The effectiveness of any phytoremediation strategy is highly dependent on many factors. The most important of these include the ability of plants to remove contaminants (Khan and Barros 2023). In this regard, a quick and easy assessment of a plant’s potential to tolerate pollutant loads is needed. One method for such assessment is to study the biochemical response of plants upon exposure to contaminants (Qurban et al. 2021; Khan et al. 2021b). In the present study, parameters such as plant photosynthetic pigments (Table 1) and biochemical profiles (protein content and enzymatic signature, Fig. 1) are pivotal for determining the capacity of selected plants for the phytoremediation of metals and metalloids. Abrupt fluctuations in photosynthetic pigments (notably Chl a, Chl b, and Car) are indications of physiological stress in plants (Nawaz et al. 2023). A stable photosynthesis profile with no phenotypic changes upon acute exposure to contaminants can be taken as an indicator of phytoremediation potential or phytotoxicity if the plant is intolerant to the provided stress (Oláh et al. 2021). Furthermore, a slower loading rate of pollutants and greater hydraulic retention time can help plants used in constructed wetlands remain stable with respect to Chl content and achieve exceptionally greater contaminant removal (Ujang et al. 2021). Similar findings were noted in the present investigation, where the 14-day BE showed highly stable Chl contents (Table 1). Plants usually show an increase in soluble protein content in a parabolic manner, which increases with an increase in the concentration of contaminants to a point where the stress is unbearable and the level decreases with any further increase in the stressor concentration (Zhao et al. 2021). Plants can undergo stress when exposed to pollutants in any environmental matrix, which can cause oxidative injury (Li et al. 2013). To combat this stress and maintain cellular balance (homeostasis), plants produce antioxidant enzymes, including APX and CAT, which play crucial roles in regulating the levels of harmful molecules within the cell (Iqbal et al. 2020). The levels of these molecules, such as superoxide (O^2−^), hydrogen peroxide (H_2_O_2_), and other stressors, increase because of HM uptake. Furthermore, upon exposure to metal(loid)s, plants are known to exhibit rapid increases in their antioxidant enzyme activities that are directly proportional to increasing pollutant concentrations and gradually reach their basal levels upon reductions in pollutant concentrations or stress (Raza et al. 2019; Saleem et al. 2023). However, the higher levels of such antioxidant activity also indicate that plant enzymatic defense fails to cope with plant stress, which can further lead to a reduction in the positive impact of enzymatic activity on plant growth, and increased production of antioxidant enzymes leads to stunted growth (Khan et al. 2021a, 2021b). Hence, with the present results, it can be inferred that at a BE of 14 days, the plants could tolerate the stress of polluted water, and the levels of CAT and APX activities were comparable to those of the controls. In terms of the activities of the two tested antioxidant enzymes (CAT and APX) in the three species in the treatment group, the catalase activity was lower than that in the control group. It has been reported that plants employ multiple response mechanisms to counteract the stress caused by metals and metalloids, ranging from the upregulation of other enzymatic antioxidant mechanisms (Singh et al. 2021; Khan et al. 2021a). In the present study, APX activity increased markedly at 7 days of BE in *P. australis* and *S. holoschoenus* compared to that in the control. However, this enzymatic response can vary with plant type, tolerance capacity, and dose of pollutants needed for upregulation, as observed in *T. angustifolia*. This upward trend in APX activity in PW samples was greater at 14 days, while at 7 days of BE, the APX activity decreased. Taken together, these results indicate that the three studied species have different physiological and molecular mechanisms to address the stress caused by water contaminated by metals and metalloids. These stresses can be beneficial to the plant, as they are also correlated with increased enzymatic activity and phenotypical observations. The consistent trends in chlorophyll content and enzyme activities from the static experiments provided valuable insights for selecting HRT durations in the flow system. This approach offered a more informed strategy than a trial-and-error method. Based on these findings, a phytoremediation experiment was designed with HRTs of 15 and 30 days.

The biomass production of a plant used for phytoremediation is a very important parameter for identifying the capacity for tolerance, as the values for fresh and dry weight of biomass (root and shoot) can be used for the computation of different indices, including water storage capacity, tissue water content, and stress tolerance (Shabir et al. 2018; Wu et al. 2021; Younas et al. 2022). Similarly, the biomass production capacity can vary significantly among plant types and species, hindering pollutant removal efficiency. One such study was conducted by Schück and Greger (2020), who reported that out of 34 studied macrophytes, only *Carex pseudocyperus* and *Carex riparia* could produce more biomass and consequently remove heavy metals better. Another study by Rai (2019) proposed a similar finding for *Eichhornia crassipes*, which performed comparatively better than *Pistia stratiotes* and *Spirodela polyrhiza* in metal- and metalloid-contaminated environments. Hence, it can be inferred that plants respond differently to biomass production and can be used as an indicator for phytoremediation. In the present study, similar results were noted (Table 2). Compared with *T. angustifolia*, both, *P. australis* and *S. holoschoenus* produced significantly more biomass and showed improved stability at the 30*-*day HRT*.* This biomass production, along with the different HRT regimens, also impacted the levels of PO_4_^−3^, NH_4_^+^, and NO_3_^−^ and the metal and metalloid contents in the water (Figs. 2 and 3). Based on these findings, it can be concluded that macrophyte plants, notably *P. australis* and *S. holoschoenus*, can perform significantly better at an HRT of 30 days or longer under similar polluted water conditions. Ujang et al. (2021) also argued that a slower loading of pollutants and a higher HRT can result in enhanced removal of pollutants, which can reach up to 100% removal if either recirculation is applied or the covered surface area of treatment by constructed wetlands can be increased when green phytoremediation technology is upgraded.

The substantial pH increase (2.7 to 7.4) and changes in the other physical and chemical parameters likely involve interactions among the aquatic plant species, growth substrate, and parent material of the gravel bed (Bolan et al. 2014). Plants can deplete acidic minerals through nutrient uptake, influencing the physical parameters of soil; for instance, nitrate uptake can lead to rhizosphere alkalization of subsurface soil (Wang et al. 2023; Gu et al. 2023). Additionally, root exudates can chelate nutrients and metal(loid)s in the rhizosphere and apoplast, generating generally fewer to nontoxic exudate complexes, leading to metal(loid) detoxification, and influencing the ability of rhizosphere microbes to consume protons (Zheng et al. 2024; Wu et al. 2019). Furthermore, gravel, depending on its parent material (e.g., limestone), can act as a cation exchanger. It can swap acidic H^+^ ions clinging to its surface for less acidic or basic cations (e.g., Ca^2+^), further lowering the H^+^ concentration and increasing the pH (Custos et al. 2020). In the present study, the translocation and compartmentalization of metals and metalloids were dose-dependent, and it is evident that the plants used can perform better phytostabilization at slower loading rates or have negative effects on plant phenotype, biomass, and phytoremediation capacity (Figs. 4 and 5, and Table 2). It is worth mentioning that a faster loading rate or lower HRT can hinder contaminant removal efficiency, which leads to more metal accumulation in the rhizosphere without proper phytostabilization, leading to higher levels of metal and metalloid that are beyond the plant’s capacity to remove and buffer (Xia et al. 2018; Li et al. 2018). The transport of metals and metalloids can follow specific or nonspecific metal transporter/ion channel pathways during the apoplast and symplastic pathways (Jamla et al. 2021). These findings highlight the complex and dynamic nature of plant metal uptake, where factors such as exposure duration and metal type significantly influence the interaction between different elements within the plant. Compared with plants exposed to shorter HRTs, plants exposed to metals for longer periods (with higher HRTs) exhibit a shift in their metal(loid) uptake strategy. It is well established that metals and metalloids are known to compete for plant uptake, which results in synergistic or antagonistic interactive effects (Kanu et al. 2019; Saleem et al. 2023). The plant’s metal transporters responsible for the uptake and stabilization of metals in plant cells become saturated due to increased metal uptake, leading to competition between different metals, particularly nonessential metals such as cadmium (Cd), arsenic (As), and lead (Pb). The exposure of plants to unwanted and xenobiotic concentrations of metal(loid)s can trigger stress responses, potentially affecting their ability to take up other elements, including essential elements such as iron and zinc (Gautam et al. 2023). Furthermore, higher loading rates or shorter retention times can lead to fluctuations in the physical and chemical parameters of water, which was also observed in the present study (Table 3, Fig. 7). Jiang and Chui (2022) also reported that the hydrological parameters of water in constructed wetlands are strongly associated with the hydraulic retention time and the hydraulic loading rate, as a higher loading rate can reduce the efficacy of phytoremediation. In accordance with this study, the present work also proposed that a greater HRT can lead to dual benefits: not only can more contaminants in water be treated but also, the toxicological profile can be significantly improved, reaching that of clean water (Fig. 6). One of the important points in this study was the potential macrophyte-assisted phytoremediation of polluted water, with reference to the *V. fischeri* bioluminescence reduction assay, as in most of the previous studies, only the reduction in the levels of pollutants in water is considered a key performance indicator, with complete neglect that the treatment of parent contamination can lead to secondary contamination of different natures (Khan et al. 2023a, b; Zheng et al. 2019). García et al. (2013) presented an initial and comprehensive study confirming that phytoremediation results in dual benefits (reduced contamination and toxicity), in which the treatment of metal-working fluid with maize-assisted phytoremediation coupled with bioaugmentation was performed, and a reduction in toxicology was confirmed using a chlorophyll fluorescence assay and a cyanobacterial bioluminescent toxicity assay. In addition, many phyco-, phyto-, and bio-toxicity assays that can be used to assess the reduction of toxicity at the endpoint after bio and phytoremediation of emerging and legacy contaminants present in different environmental matrices have been reported in the published literature (Hussain et al. 2021b; de la Parra et al. 2022). For the continuation of research in this phytoremediation line, it is highly recommended that speciation of pollutants using sequential extraction be introduced to assess the changes in contamination between bioavailable, organic matter-bound, suspended, ion exchangeable, oxidizable, and residual (unavailable) pollutants. Furthermore, the impact of dosing rates should be evaluated in static and dynamic wetland mesocosm systems to understand the concept of acute toxicity and gradual acclimatization of macrophytes in contaminated environments. Furthermore, investigating metal removal mechanisms beyond plant uptake is crucial. This includes examining precipitation processes triggered by plant exudates, adsorption onto roots and the substrate, and other physicochemical interactions in the root zone. These factors can contribute to increased removal at higher HRTs without necessarily reflecting greater metal extraction and internalization by aquatic plants. Lastly, previous research has suggested that integrating phytoremediation with other methods (biological, chemical, physical, and electrical methods applied individually or in combination) can achieve efficient remediation in a shorter timeframe through synergistic effects (Liu et al. 2020); however, further research is needed in this area.

## Conclusion

For the remediation of metal(loid)s in water, the use of macrophyte-assisted phytoremediation is a promising biotechnological intervention. Although all three plant species (*Phragmites australis*, *Scirpus holoschoenus*, and *Typha angustifolia*) tolerated the contaminated water, *P. australis* and *S. holoschoenus* exhibited superior performance based on stable photosynthetic pigment production and enzymatic activity (APX and CAT), particularly at longer treatment times (30 days). This indicates that their ability to maintain physiological functions is crucial for growth and contaminant processing under stress. Importantly, this study highlights that achieving truly holistic phytoremediation goes beyond pollutant reduction. Furthermore, this study explored the ability of these three macrophytes to clean metal- and metalloid-contaminated groundwater after exposure to 15- and 30-day HRTs. Plant selection, water properties, and contaminant levels all significantly impact phytoremediation efficiency, which was also evident in the present trials. All three species tolerated the contaminated water, with *P. australis* and *S. holoschoenus* showing better growth with longer treatment times (30 days) leading to greater removal and lower plant tissue accumulation, indicating better contaminant management. Even though the reduction in pollutant load (legacy and emerging) in water and other environmental matrices is a key performance indicator for phytoremediation, it should be noted that for holistic phytoremediation, the endpoint toxicity in the treated matrices must be lower than that of no treatment. It can also be concluded that for dually efficient application, individual phytoremediation should be carried out with a greater HRT. For wider application, future phytoremediation research should focus on improving efficiency by identifying and developing new plant cultivars, understanding underlying mechanisms, and developing integrated systems.

## Supplementary Information

Below is the link to the electronic supplementary material.

Supplementary file1 (DOCX 3571 KB)
