# Supplementary material for: Macrophyte assisted phytoremediation and toxicological profiling of metal(loid)s polluted water is influenced by hydraulic retention time
Source: Environ Sci Pollut Res Int. 2024 Jun 19;32(28):16760–79. doi: 10.1007/s11356-024-33934-2 (PMC12325410; doi:10.1007/s11356-024-33934-2)
Supplement: Supplementary file 1 — Supplementary file1 (DOCX 3571 KB) [file 11356_2024_33934_MOESM1_ESM.docx]

**Supplementary information**

Macrophyte assisted phytoremediation and toxicological profiling of metal(loid)s laden ground water is influenced by hydraulic retention time.

Aqib Hassan Ali Khan^a^, Alberto Soto-Cañas^a^, Carlos Rad^a^, Sandra Curiel-Alegre^a,b^, Carlos Rumbo^a^, Blanca Velasco-Arroyo^c^, Herwig De Wilde^d^, Alfredo Pérez-de-Mora^e^, Rocío Barros^a^

*^a^International Research Center in Critical Raw Materials for Advanced Industrial Technologies (ICCRAM), University of Burgos, Centro de I+D+I. Plaza Misael Bañuelos s/n. 09001, Burgos, Spain*

*^b^Research Group in Composting (UBUCOMP), University of Burgos, Faculty of Sciences, Plaza Misael Bañuelos s/n, 09001, Burgos, Spain*

*^c^Department of Biotechnology and Food Science, University of Burgos, Plaza Misael Bañuelos, s/n. 09001, Burgos, Spain*

*^d^TAUW België nv, Dept. of Soil and Groundwater, Waaslandlaan 8A3, 9160 Lokeren, Belgium*

*^e^TAUW GmbH, Dept. of Soil and Groundwater, Landsbergerstr. 404, 81241 Munich, Germany*

***Corresponding authors:** [rbarros@ubu.es](mailto:rbarros@ubu.es), [ahkhan@ubu.es](mailto:ahkhan@ubu.es)


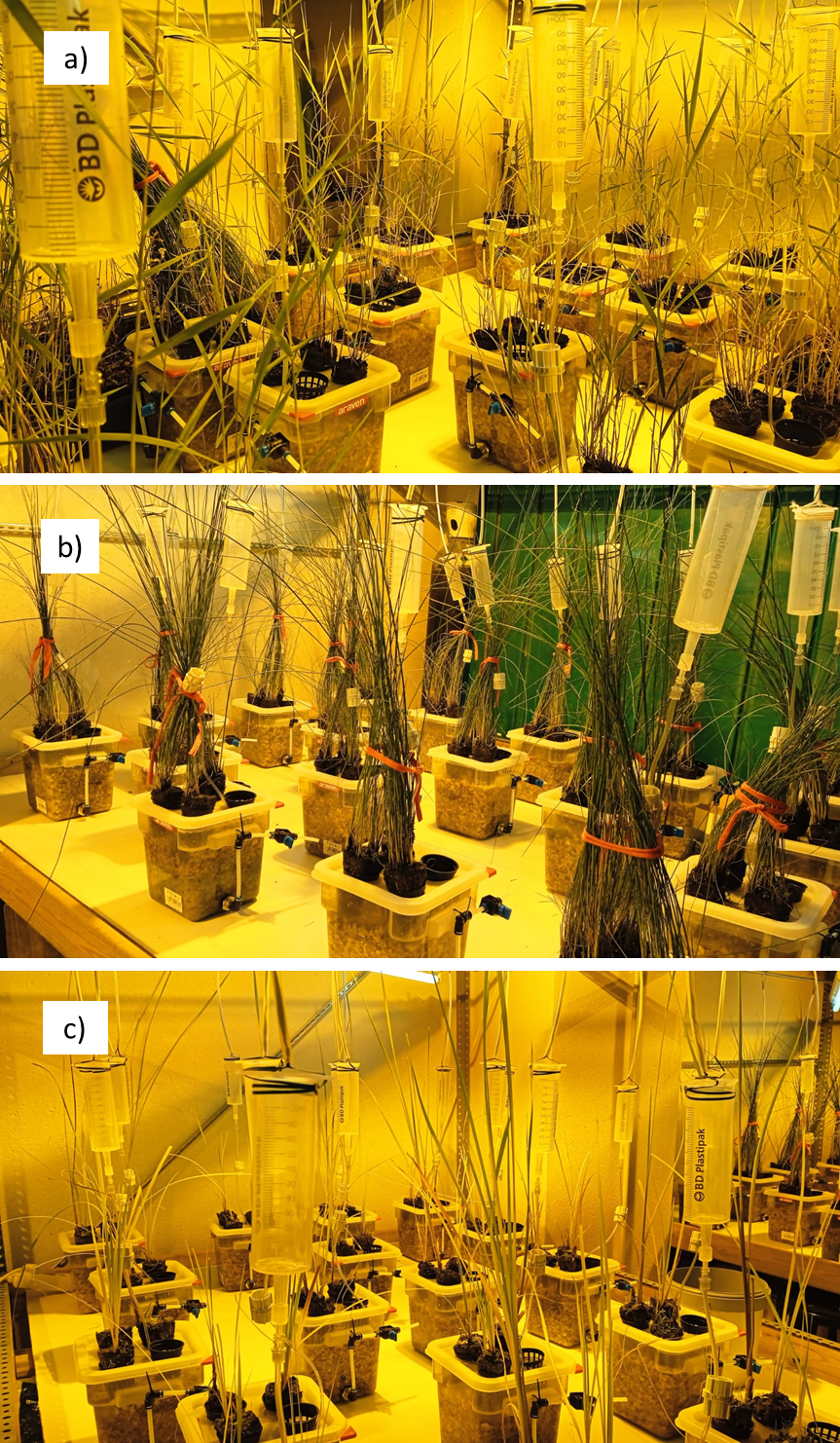


**Supplementary Figure 1.** Plant canopy produced by the selected macrophytes upon exposure to polluted water at 15- and 30-days HRT. Images of canopy presented here as a) *P. australis*, b) *S. holoschoenus,* and c) *T. angustifolia.*

**Supplementary Table 1.** Summary of Network produced with different HRT for metal and metalloid relation.

| **HRT** | **Number of nodes** | **Number of non-zero edges** | **Sparsity** |
| --- | --- | --- | --- |
| 30 days | **7** | **17 / 21** | **0.190** |
| 15 days | **7** | **17 / 21** | **0.190** |
|  |  |  |  |

**Supplementary Table 2.** Weights matrix for plant metal and metalloid uptake with 30 days HRT

|  | | | | | | | | | | | | | | | | |
| --- | --- | --- | --- | --- | --- | --- | --- | --- | --- | --- | --- | --- | --- | --- | --- | --- |
|  | | **Network** | | | | | | | | | | | | | | |
| **Variable** | | **Fe** | | **Ni** | | **Zn** | | **Cu** | | **As** | | **Cd** | | | **Pb** | |
| Fe |  | 0.000 |  | 0.024 |  | 0.242 |  | 0.565 |  | 0.758 |  | -0.446 |  | 0.082 | |  |
| Ni |  | 0.024 |  | 0.000 |  | 0.698 |  | 0.000 |  | 0.087 |  | 0.000 |  | 0.005 | |  |
| Zn |  | 0.242 |  | 0.698 |  | 0.000 |  | 0.257 |  | -0.134 |  | 0.276 |  | 0.000 | |  |
| Cu |  | 0.565 |  | 0.000 |  | 0.257 |  | 0.000 |  | -0.372 |  | 0.445 |  | -0.264 | |  |
| As |  | 0.758 |  | 0.087 |  | -0.134 |  | -0.372 |  | 0.000 |  | 0.000 |  | 0.396 | |  |
| Cd |  | -0.446 |  | 0.000 |  | 0.276 |  | 0.445 |  | 0.000 |  | 0.000 |  | 0.648 | |  |
| Pb |  | 0.082 |  | 0.005 |  | 0.000 |  | -0.264 |  | 0.396 |  | 0.648 |  | 0.000 | |  |
|  | | | | | | | | | | | | | | | | |

**Supplementary Table 3.** Weights matrix for plant metal and metalloid uptake with 15 days HRT

|  | | | | | | | | | | | | | | | | | |
| --- | --- | --- | --- | --- | --- | --- | --- | --- | --- | --- | --- | --- | --- | --- | --- | --- | --- |
|  | | **Network** | | | | | | | | | | | | | | | |
| **Variable** | | **Fe** | | **Ni** | | **Zn** | | **Cu** | | **As** | | | **Cd** | | | **Pb** | |
| Fe |  | 0.000 |  | 0.000 |  | -0.206 |  | 0.454 |  | 0.700 |  | -0.156 | |  | 0.030 | |  |
| Ni |  | 0.000 |  | 0.000 |  | 0.692 |  | 0.569 |  | 0.004 |  | 0.206 | |  | 0.000 | |  |
| Zn |  | -0.206 |  | 0.692 |  | 0.000 |  | 0.000 |  | 0.143 |  | 0.171 | |  | 0.048 | |  |
| Cu |  | 0.454 |  | 0.569 |  | 0.000 |  | 0.000 |  | 0.000 |  | 0.077 | |  | 0.264 | |  |
| As |  | 0.700 |  | 0.004 |  | 0.143 |  | 0.000 |  | 0.000 |  | -0.255 | |  | -0.262 | |  |
| Cd |  | -0.156 |  | 0.206 |  | 0.171 |  | 0.077 |  | -0.255 |  | 0.000 | |  | -0.309 | |  |
| Pb |  | 0.030 |  | 0.000 |  | 0.048 |  | 0.264 |  | -0.262 |  | -0.309 | |  | 0.000 | |  |
|  | | | | | | | | | | | | | | | | | |
